# Evaluation of autonomic involvement in Parkinson’s disease using pupillometry

**DOI:** 10.1007/s10072-025-08182-y

**Published:** 2025-04-15

**Authors:** Dilek İşcan, Ceren Türkoğlu, Elif Arslan

**Affiliations:** 1 Faculty of Medicine, Department of Neurology, Niğde Ömer Halisdemir University, Niğde, 51240 Turkey; 2 Faculty of Medicine, Department of Ophthalmology, Lokman Hekim University, Ankara, Turkey; 3 Department of Ophtalmology, Ulucanlar Eye Education and Research Hospital, Ankara, Turkey

**Keywords:** Parkinson’s disease, Dysotonomia, Pupillometry, Pupillary light reflex

## Abstract

**Introduction:**

The pupillary light reflex (PLR) indicates the function of the autonomic nervous system, which causes the pupil to constrict and dilate. Evaluation of the PLR determines the parasympathetic and sympathetic balance. We aimed to demonstrate autonomic changes by pupillometry in Parkinson’d disease (PD) and to investigate the relationship between the changes and motor stage and levodopa equivalent dose (LEDD).

**Method:**

Static pupillometry measurement was performed at scotopic, mesopic and photopic settings. Dynamic pupillometry measurement was performed at 500 lux illumination and pupil diameter was recorded. Static and dynamic pupillometry parameters of Parkinson’s disease patients and healthy control group of similar age and gender were compared. The relationship between Unified Parkinson’s Disease Rating Scale (UPDRS)-motor, modified Hoehn and Yahr (mHYE) and levodopa equivalent daily dose (LEDD) and pupillometry parameters were analysed.

**Results:**

In static pupillometry, mesopic pupil diameter was significantly lower in PD patients (*P* = 0.04). In dynamic pupillometry, pupil diameter was lower and mean pupil dilation rate was lower in the PD group at 18 seconds. There was a significant negative correlation between mean pupil dilatation velocity and mHYE, UPDRS-motor and LEDD. The mean pupil dilatation velocity was statistically significantly lower in patients not receiving dopaminergic treatment.

**Conclusion:**

Changes in pupillometry values in PD have emphasized that the autonomic nervous system is affected and the parasympathetic nervous system was found to be correlated with the motor involvement of the disease.

## Introduction

Parkinson’s disease (PD) is the second most prevalent neurodegenerative disorder after Alzheimer’s disease and is characterized by both motor and nonmotor symptoms, including autonomic dysfunction. Autonomic impairments in PD encompass gastrointestinal disturbances, cardiovascular irregularities, urinary dysfunction, sexual dysfunction, thermoregulatory abnormalities, and pupillomotor and lacrimal disturbances [1]. Some studies suggest that autonomic symptoms such as constipation, orthostatic hypotension (OH), urinary dysfunction, erectile dysfunction, and pure autonomic failure may serve as prodromal indicators of PD [2].

Ocular and visual impairments are more prevalent in patients with PD than in the general population, either due to disease-associated pathologies or the side effects of medications used in PD treatment. These disturbances, which include dry eye, diplopia, glaucoma, and glaucoma-like visual abnormalities, contrast and color vision impairment, visuospatial and visual-perceptual deficits, and visual hallucinations, are clinically significant for disease management [3]. Studies have reported alterations in pupil reactivity in patients with PD, such as increased pupil diameters, asymmetry in pupil size following light adaptation, prolonged light reflex latency, extended constriction time, and reduced contraction amplitude [4]. While most of these abnormalities are linked to dopamine depletion in the retina and central nervous system, defects in pupil reactivity are primarily associated with dysautonomia [5].

The pupillary light reflex (PLR) regulates pupil constriction and dilation, influencing image quality and responsiveness to light, and serves as an indicator of autonomic nervous system function. The dilator and sphincter muscles, which control PLR, are innervated by the sympathetic and parasympathetic nervous systems, respectively [6]. The parasympathetic pathway mediates miosis, while the sympathetic pathway governs mydriasis [7]. In PD, pupillomotor and lacrimal abnormalities are common due to ocular autonomic nervous system dysfunction, affecting both motor and nonmotor aspects of the disease [8]. Changes in PLR may be associated with pathologies affecting neural structures such as the Edinger–Westphal nucleus and the upper cervical ganglion [9].

PLR assessment is a noninvasive, rapid, and reliable method for evaluating parasympathetic and sympathetic balance, making it a valuable diagnostic tool for neurological disorders. This study employed pupillometry to examine the relationship between pupillary changes, PD motor staging, and levodopa equivalent daily dose (LEDD). We aimed to assess its potential utility as a prodromal biomarker or a staging tool for PD and its feasibility for routine clinical use. To the best of our knowledge, this is the largest study to date examining pupillary autonomic dysfunction in PD, including a control group of healthy individuals. Our investigation employs a novel systems-based approach to explore autonomic dysfunction in PD.

## Materials and methods

This study included patients diagnosed with idiopathic PD who exhibited no clinical evidence of autonomic dysfunction and an age- and sex-matched healthy control group. Participants were recruited from the Neurology Outpatient Clinic of Niğde Training and Research Hospital between May and August 2023. Patients without symptoms of constipation, urinary incontinence, hand and foot discoloration, orthostasis, abnormal sweating, sialorrhea, dry mouth, or dry eyes were considered to have no clinical autonomic involvement. The study was approved by the local ethics committee (protocol no. 2023/3) and conducted in accordance with the Declaration of Helsinki. Written informed consent was obtained from all participants.

Exclusion criteria included the presence of additional neurological or ophthalmic diseases, systemic conditions associated with autonomic dysfunction (e.g., diabetes mellitus or chronic renal failure), glaucoma or corneal disease affecting pupillometry, a history of ocular surgery, and the use of anticholinergic, cholinergic, or adrenergic medications for PD treatment.

### Neurological examination

PD was diagnosed based on the United Kingdom PD Society Brain Bank Clinical Diagnostic Criteria [10]. A comprehensive medical history was obtained, and neurological examinations were conducted. Patients with secondary parkinsonism were excluded based on their medical history, clinical examination, and neuroimaging findings. The disease onset time, comorbidities, and current medications were documented. Antiparkinsonian medications were converted to LEDD [11].

### Unified PD rating scale–motor assessment

Patients were clinically assessed using the motor section of the Unified PD Rating Scale (UPDRS). The UPDRS is designed to evaluate motor function, cognitive and emotional state, and activities of daily living in PD patients. The third section specifically assesses motor function [12]. In this study, only part 3 of the UPDRS was applied, and assessments were conducted during the “On” period of PD treatment.

### Modified Hoehn–Yahr scale

The Hoehn and Yahr scale, originally developed in 1967 by Margaret Hoehn and Melvin Yahr, is an eight-stage system used to classify PD severity, with higher stages indicating increased symptom severity [13].

### Ophthalmological examination and pupillometry

A complete ophthalmological evaluation, including an anterior segment examination and fundoscopy, was performed for all participants. Best-corrected visual acuity, assessed using the Snellen chart, was converted to LogMAR values.

Pupillometry measurements were conducted using the Sirius 3D rotational Scheimpflug camera topography system (Costruzione Strumenti Oftalmici, CSO, Italy) by the same examiner in a dark room. To minimize the influence of age, sex, and circadian variations on pupil size, measurements were taken at a standardized time interval (12:00–13:30) in age- and sex-matched groups. For patients on levodopa therapy, pupillometry was performed 1 h after drug administration, within the same time window (12:00–13:30). Participants were instructed to fixate on a target with both eyes open. Measurements were taken first from the right eye, followed by the left eye, with brief intervals allowing for blinking. Only right eye values were included in the study in case of potential small anisocoria, which may cause statistical inaccuracies.

Static pupillometry measurements were performed after 20 min of dark adaptation under three different lighting conditions: 0.4 lx (scotopic), 4 lx (mesopic), and 40 lx (photopic), following the device’s protocol. Each measurement was repeated three times per lighting condition. Dynamic measurements commenced when the disk was illuminated at 500 lx, with pupil diameter recorded at 0 and 18 s after the light was turned off. The average pupil dilation velocity was calculated as the difference in pupil width between 0 and 18 s divided by 18 (mm/s).

### Statistical analysis

Statistical analysis was conducted using Statistical Package for the Social Sciences (SPSS) software version 26 (SPSS Inc., Chicago, Illinois). Data were summarized as mean ± standard deviation and percentage values. The Kolmogorov–Smirnov test was used to assess the normality of data distribution. Parametric tests were applied to normally distributed data, while nonparametric tests were used for nonnormally distributed data. The independent samples t test was used for parametric comparisons between two independent groups, whereas the Mann–Whitney U test was applied for nonparametric comparisons. Correlations between variables were analyzed using the nonparametric Spearman correlation test. A significance level of *p* < 0.05 was considered statistically significant.

## Results

A total of 152 right eyes from 152 participants were analyzed. Among the participants, 55.3% (*n* = 84) were male, and 44.7% (*n* = 68) were female, with a mean age of 69.45 ± 6.40 years (range: 55–85 year). Participants were divided into two groups: Group I (patients with PD) and Group II (healthy controls). No significant differences were found between the groups regarding age and gender (*p* = 0.07 and *p* = 0.5, respectively). The demographic characteristics of the participants are presented in Table 1.

**Table 1:** Demographic characteristics of the participants

	Group I (*n* = 76)	Group II (*n* = 76)	All participants (*n* = 152)
Age	70.39 ± 8.24	68.50 ± 3.58	69.45 ± 6.40
Male	57.9% (*n* = 44)	52.6% (*n* = 40)	55.3% (*n* = 84)
Female	42.1% (*n* = 32)	47.4% (*n* = 36)	44.7% (*n* = 68)

The mean disease duration of Group I was 3.97 ± 4.34 years. The mean UPDRS-motor score was 11.39 ± 12.87 (range: 8–41), the mean modified Hoehn–Yahr stage (mHYE) was 1.85 ± 0.63 (range: 1–4), and the mean LEDD was 519.54 ± 355.80 mg.

In static pupillometry, no significant differences were observed between groups in scotopic or photopic pupil diameters (*p* > 0.05). However, the mesopic pupil diameter was significantly lower in patients with PD compared with controls (*p* = 0.04). In dynamic pupillometry, a significant difference was no found in pupil diameter at 0. second, with patients with PD exhibiting significantly reduced diameters at 18. seconds (*p* = 0.04). Static and dynamic pupillometry values are summarized in Table 2.

**Table 2:** Dynamic and static pupillometry values

Features		Group I (mm)	Group II (mm)	*P*
Static pupillometry values	Scotopic diameter	4.61 ± 0.99	4.81 ± 0.67	*0.1*
	Mesopic diameter	4.31 ± 0.94	4.60 ± 0.72	***0.04***
	Photopic diameter	3.80 ± 0.90	4.01 ± 0.53	*0.7*
Dynamic pupillometry values	0.sec.	3.24 ± 0.78	3.24 ± 0.58	*0.9*
	18.sec.	4.36 ± 0.92	4.97 ± 0.88	***< 0.001***
The mean pupil dilation velocity (mm/s, 0–18 s.)		0.06 ± 0.04	0.10 ± 0.04	***< 0.001***

Independent-Samples T Test. Values are presented as number or mean and standard deviation

Analysis of pupil dilation over the 0- to 18-s interval revealed a statistically significant reduction in pupil dilation velocity in patients with PD compared with controls (*p* < 0.001), with slower dilation observed in the PD group (Group I).

Multivariate correlation analysis evaluated the associations between static and dynamic pupillometry values and UPDRS-motor scores, mHYE stages, and LEDD in PD patients (Group I). Significant negative correlations were identified between mean pupil dilation velocity and UPDRS-motor score (*r* = − 0.47, *p* = 0.001), mHYE stage (*r* = − 0.21, *p* = 0.04), and LEDD (*r* = − 0.25, *p* = 0.03). These findings are presented in Table 3.

**Table 3:** The multivariate correlations between dynamic - static pupillometry values and UPDRS-motor score, mHYE, LEDD

Variables		SD	MD	PD	PDV (mm/s, 0–18 s.)	UPDRS-motor score	mHYE	LEDD
SD	Correlation *P* value N		0.91** < 0.01 76	0.78** < 0.01 76	0.094 0.21 76	0.14 0.20 40	0.02 0.44 66	-0.1 0.47 62
MD	Correlation *P* value N			0.90** < 0.01 76	0.14 0.12 76	0,18 0,13 40	-0.009 0.47 66	-0,16 0.11 62
PD	Correlation *P* value N				0.89 0.22 76	0.90 0.29 40	0.01 0.46 66	-0.16 0.11 62
PDV (mm/s, 0–18 s.)	Correlation *P* value N					-0.47** 0.001 40	-0.21* 0.04 66	-0.25* 0.03 62
UPDRS-motor	Correlation *P* value N						0.58** < 0.001 40	-0.21 0.12 32
mHYE	Correlation *P* value N							0.34** 0.006 54
LEDD	Correlation *P* value N							

Multivariate correlation analyses, SD: Sctopic diameter, MD: Mesopic diameter, PD: Photopicdimeter, PDV: The mean pupil dilation velocity, UPDRS-motor score: United Parkinson’s Disease Rating Scale-motor score, mHYE: modifiye Hoehn and Yahr Scale, LEDD: levodopa equivalent daily dosage, **. Correlation is significant at the 0.01 level, *. Correlation is significant at the 0.05 level

Among the PD patients, 60 were receiving dopaminergic treatment (levodopa and dopamine agonists), while 16 were not. The mean pupil dilation velocity was significantly lower in patients who were not receiving dopaminergic treatment (*p* = 0.01).

## Discussion

This study found that mesopic pupil diameter in static pupillometry as well as pupil diameter at 18 s and mean pupil dilation rate in dynamic pupillometry were significantly lower in patients with PD. Alterations in dynamic pupillometry indicate sympathetic nervous system involvement. Given the sympathomimetic effects of dopaminergic agents—commonly used by PD patients—sympathetic effects may be more pronounced when patients are off medication. Additionally, negative correlations were observed between dynamic pupillometry changes from 0 to 18 s and UPDRS-motor scores, mHYE and LEDD, suggesting increased sympathetic nervous system involvement as PD progresses. The significantly lower mean pupil dilation velocity in patients not receiving dopaminergic treatment may be attributed to the sympathomimetic effects of these drugs.

A study by Giza et al. [14] comparing PLR parameters between patients with PD and controls demonstrated a significant increase in latency and a decrease in the poststimulus radius (R2), initial radius (R1) amplitude, maximum speed, and maximum acceleration. However, no significant differences were noted in R1 or the minimum radius (R2). These findings align with the slower dilation rate observed in dynamic parameters in our study, though no significant differences were detected in static parameters except for mesopic diameter.

Similarly, Fotiou et al. [15] conducted a pupillometric examination in patients with hemiparkinsonism and found a significant decrease in maximum speed and acceleration compared with controls. Their results support our findings, which indicate a slower dilation rate in the PD group.

Bartosova et al. [16] identified a significant relationship between levodopa dosage and pupillometric parameters through nonlinear regression analysis, showing correlations between both morning and total daily levodopa doses and maximum values. Conversely, Gaynes et al. [17] found no correlation between PLR and disease severity or levodopa dosage. Giza et al. [14] also explored the relationship between pupillometry parameters and clinical features such as disease duration, stage, and UPDRS-motor scores but found no significant correlation. In contrast, our study identified a significant negative correlation between mean pupil dilation velocity and both LEDD and UPDRS-motor scores.

In the study by You et al. [6], pupillometry values were compared between early- and advanced-stage PD patients. Their findings indicated that PLR changes with disease progression, as significantly higher pupil diameters were observed in early-stage PD compared with late-stage PD. Additionally, constriction speed and maximum constriction speed were significantly affected by disease progression, whereas variables such as minimum diameter and dilation rate showed no significant differences.

Feigl et al. [18] examined the relationship between melanopsin cell dysfunction and sleep disturbances in PD, categorizing patients based on disease severity. However, they did not observe differences in PLR among groups.

In a study by Tsitsi et al. [19], which included healthy controls and PD patients with and without OH, the researchers reported lower pupil diameters in the PD group following long-flash stimuli, though the difference was not statistically significant. PD patients with OH exhibited slower and less effective pupil dynamics in both short- and long-flash stimuli.

This study has several limitations. It was a single-center, cross-sectional study that did not classify PD patients based on tremor or akinesia dominance, compare early and late disease stages, or account for differences in therapy regimens. Additionally, patients were assessed while receiving their current treatments, and no comparisons were made between treatment-naïve and treated groups. Autonomic symptoms were determined solely through patient history, and nonmotor symptoms beyond autonomic involvement were not assessed. Moreover, no comparisons were conducted using alternative autonomic assessment methods. Future studies should include pupillometry measurements at specific time intervals within a larger cohort to analyze the relationship between pupillometry parameters and disease progression.

## Conclusion

Although autonomic involvement in PD is well recognized, its clinical significance may be overlooked if patients do not report symptoms. Implementing a simple, cost-effective, brief, and noninvasive tool into routine clinical assessments could enhance the identification and management of autonomic dysfunction. While pupillometry is not yet a standard component of PD evaluation, further comprehensive studies may support its integration into clinical practice.
